# Modeling tau aggregation through optogenetics

**DOI:** 10.1002/alz.087377

**Published:** 2025-01-03

**Authors:** Marcos Schaan Profes, Lyucheng Zou, Kurt W. Farrell, John F. Crary

**Affiliations:** ^1^ Department of Pathology, Icahn School of Medicine at Mount Sinai, New York, NY USA; ^2^ Icahn School of Medicine at Mount Sinai, New York, NY USA

## Abstract

**Background:**

The molecular etiology of tau‐derived neurodegeneration remains poorly understood, reflected in the low success rate of clinical trials. Hence, aquiring a better understanding the molecular basis of tauopathies is a critical need.

**Objective:**

To develop a versatile and reproducible system to study tau aggregation with high spatiotemporal control through optogenetics that will aid in investigating the differences in tau aggregation kinetics, the burden the burden of tau isoforms, and mutations and that will be suitable for high‐throughput analysis of tauopathy‐related mechanisms.

**Method:**

We engineered an optogenetic cellular model using the CRY2 protein that when light‐activated enables spatiotemporal control over tau aggregation. A panel of CRY2‐tau constructs expressing all six neuronal isoforms and known mutations was created to assess aggregation kinetics and cellular burden due to imbalances in tau proteoforms. Tauopathy‐associated factors will be assayed with biochemistry, and challenge optogenetic tau aggregation with their overexpression and knockdown. Experiments in patient‐derived human induced pluripotent stem cell models will be performed supporting the project’s translational nature.

**Result:**

HEK293 and SH‐SY5Y cells expressing our constructs showed mCherry stable tau inclusions after light stimulation, which are not present when the backbone alone is transfected and are less frequent without light stimulus. These inclusions where positive for phosphorylation markers consistent with Alzheimer disease when assessed by immunocytochemistry. Western blotting analysis confirmed the presence of high molecular weight tau molecules consistent with dimmers and higher aggregates and live‐imaging assessments of different tau proteoforms suggest different CRY2‐tau inclusion formation kinetics.

**Conclusion:**

These data support the feasibility of generating tau aggregates with pathological features in our cellular system. This approach has the potential to enhance the screening process of possible tau aggregation modulators allowing analysis in real time of tau aggregation in a precise manner with high spatiotemporal resolution while. This model may r be amenable to drug screening.

